# Identifying target behaviors for weight management interventions for women who are overweight during pregnancy and the postpartum period: a qualitative study informed by the Behaviour Change Wheel

**DOI:** 10.1186/s12884-021-03689-6

**Published:** 2021-03-11

**Authors:** Johanna Saarikko, Hannakaisa Niela-Vilén, Amir M. Rahmani, Anna Axelin

**Affiliations:** 1 Department of Nursing Science, University of Turku, Turku, Finland; 2 Department of Obstetrics and Gynaecology, Turku University Hospital and Faculty of Medicine, University of Turku, Turku, Finland; 3 School of Nursing, University of California, Irvine, USA; 4 Department of Computer Science, University of California, Irvine, USA

**Keywords:** Pregnancy, Overweight, Obesity, Weight-management, Technology, Communication, Experience, Qualitative research, Perinatal care

## Abstract

**Background:**

Maternal overweight is increasing, and it is associated with several risk factors for both the mother and child. Healthy lifestyle behaviors adopted during pregnancy are likely to impact women’s health positively after pregnancy. The study’s aim was to identify and describe weight management behaviors in terms of the Capability, Opportunity and Motivation Behaviour (COM-B) -model to target weight management interventions from both the perspectives of women who are overweight and maternity care professionals.

**Methods:**

This qualitative, descriptive study was conducted between 2019 and 2020. Individual interviews with pregnant and postpartum women who were overweight (*n* = 11) and focus group interviews with public health nurses (*n* = 5) were undertaken in two public maternity clinics in Southwest Finland. The data were analyzed using deductive content analysis consistent with the COM-B model.

**Results:**

In the capability category, the women and the public health nurses thought that there was a need to find consistent ways to approach overweight, as it had often become a feature of the women’s identities. The use of health technology was considered to be an element of antenatal care that could be used to approach the subject of weight and weight management. Smart wearables could also support an evaluation of the women’s lifestyles. The opportunity category highlighted the lack of resources for support during perinatal care, especially after birth. Both groups felt that support from the family was the most important facilitating factor besides motivation. The women also expressed a conflict between pregnancy as an excuse to engage in unhealthy habits and pregnancy as a motivational period for a change of lifestyle. Furthermore, the women wanted to be offered a more robust stance on weight management and discreet counseling.

**Conclusions:**

Our findings offer a theoretical basis on which future research can define intervention and implementation strategies. Such interventions may offer clear advice and non-judgmental support during pregnancy and after delivery by targeting women’s capabilities, opportunities, and motivation. Health technology could be a valuable component of intervention, as well as an implementation strategy, as they provide ways during maternity care to approach this topic and support women.

## Background

Maternal overweight, defined as a body mass index (BMI) of 25 or higher before pregnancy, increases the risks for the mother and her infant [1–3]. Obesity among women of reproductive age is increasing globally, especially in high-income countries [4]. In Finland, 36% of pregnant women are overweight, and 14% are obese; these numbers have risen over time [5]. Pregnant women visit maternity clinics frequently, so the public health nurses at these clinics play a key role in supporting pregnant women with weight management [6]. In addition, women might be more open to adopting health behaviors during pregnancy [7].

Women who are overweight and maternity clinic nurses are reluctant or find it difficult to discuss weight and weight management during maternity care [8, 9]. Many women lack the knowledge and skills to manage their weight effectively. However, research has shown that women who are overweight want information about the risks of obesity, and to be given more information and support for weight management during pregnancy and the postnatal period [9–12]. Furthermore, researchers have identified a range of effective interventions for weight management during pregnancy and the postpartum period, but these interventions have proved difficult to implement in clinical practice [13, 14].

There is a lack of effective strategies for supporting weight management in maternity care [15]. Health technology applications that support weight management could be useful alternatives to standard practices [16, 17] and would provide the possibility for example, of continuously monitoring pregnant women’s physiological parameters. Real-time information between scheduled appointments could support weight management counseling in maternity care [18]. Health technology could also provide solutions to the difficulties experienced in the implementation of effective weight-management interventions.

Although several previous studies have examined the experiences of women who were overweight or obese during pregnancy, there is a need to identify more closely the behaviors of these women through focusing on problematic areas and choosing effective implementation strategies [19, 20]. Target behaviors should be clarified in order to tailor interventions to the identified needs of the women. To address this implementation barrier, the following theoretical frameworks were used to gain a rigorous understanding of the needs for effective weight-management support: the Behaviour Change Wheel (BCW), the Capability, Opportunity and Motivation Behaviour (COM-B) model, and the Theoretical Domains Framework (TDF).

The aim of this study was to identify and describe weight management behaviors in terms of the COM-B -model from the perspectives of maternity care professionals and women who are overweight or obese.

## Methods

### Design and setting

This study is a part of multiphase research project aimed at tailoring and implementing an evidence-based weight management intervention for pregnant women who are overweight. A qualitative, descriptive study design was used to investigate perceptions of weight management support among pregnant and postpartum women who are overweight. In Finland, women receive free care at maternity clinics located in primary health care centers run by registered public health nurses or registered midwives. Semi-structured individual and focus group interviews were undertaken in two public maternity clinics located in four municipalities in the Hospital District of Southwest Finland. The study was reported according to the Consolidated Criteria for Reporting Qualitative Research checklist.

### Participants

A purposive sample of women who are overweight and public health nurses were recruited into the study between April 2019 and January 2020. The women were eligible if they were 1) pregnant or had delivered their baby in the past six months, 2) ≥ 18 years of age, and 3) overweight (BMI ≥ 25). The public health nurses identified the eligible women and informed them about the study. After providing oral and written information, the public health nurses asked the women for their permission for the researcher to contact them as potential participants. All the public health nurses (*n* = 8) working at the selected maternity and child health clinics were eligible for participation in the study. The researcher contacted the public health nurses via e-mail and scheduled meetings after receiving approval from the Nursing Director of the health center.

### Theoretical approach

**Fig. 1:**
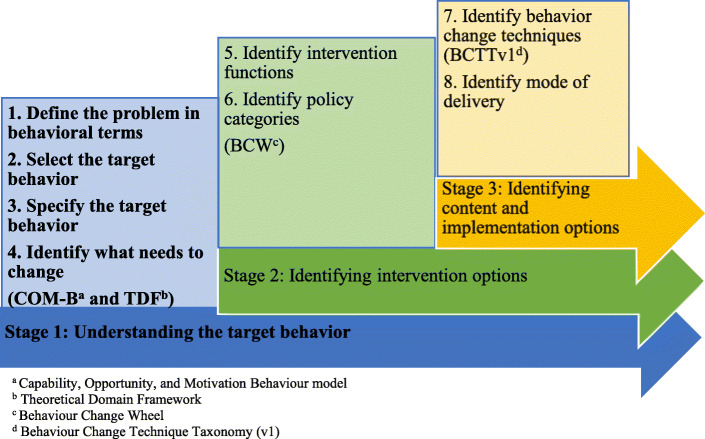
Development of an intervention using the BCW [20]

The BCW was used as a theoretical framework to understand and identify the cognitive, affective, social, and environmental influences of overweight and obesity during pregnancy on behavior [20–22]. The BCW consists of three core components: 1) a model of behavior that includes physical and psychological capability, physical and social opportunity, and automatic and reflective motivation (i.e., the COM-B-model); 2) intervention functions; and 3) policy categories, which are decisions made by authorities that enable intervention functions to occur. According to the BCW model, behavior is an interactive system involving capability, opportunity, and motivation that can be changed by the intervention functions [20]. The TDF, a meta-framework incorporating 33 psychology theories and over 128 behavioral change constructs, can be used to expand on the COM-B model’s components in order to identify target behaviors [20]. The TDF has 14 theoretical domains related to the capability, opportunity, and motivation of individuals to support behavior change [22]. Compatibility with the TDF increases the COM-B model’s theoretical thoroughness because the TDF has been widely used and validated in studies identifying the determinants of behavior change [23–25]. According to the BCW, intervention development processes are categorized into three stages and are further subdivided into eight steps, as depicted in Fig. 1. This study focuses on Stage 1, which prepares the basis for understanding the target behavior [21].

### Interview guides

Semi-structured interview guides, informed by the BCW theory, for both women who were overweight and the public health nurses, were developed by the researchers (JS, HN-V and AA). Both guides included themes about weight-management support, the barriers and facilitators to achieving weight-management interventions, as well as utilizing technology in weight-management support.

### Data collection

The women who were overweight were initially approached by the public health nurses and contacted by telephone; they were invited to participate in an interview at a time convenient to them. The public health nurses willing to participate were contacted by e-mail or telephone in order to arrange a scheduled time for the focus group interviews. The first author (JS) informed all the participants about the purpose of the study and obtained their written informed consent. The first author (JS) conducted 11 individual interviews with pregnant or postpartum women, and two researchers (JS and HN-V) conducted two focus group interviews with the public health nurses. The focus group interviews lasted between 60 and 90 min, and the individual interviews lasted between 35 and 75 min. All the interviews were audio-recorded, and field notes were made. Researcher (JS) collected the background information from the participants using a questionnaire.

### Analysis

The audio-recorded interviews were transcribed verbatim, and the data were transferred to an NVivo 12Plus (QSR International Pty Ltd. Version 12, 2018) by the first author (JS). Deductive content analysis was conducted to organize the data into a systematically structured format [26]. The first author (JS) conducted the initial analysis by reading the transcripts and labeling codes using a deductive framework composed of the COM-B model and TDF [21]. The research team members (HN-V & AA) convened and compared the codes applied to the transcripts to avoid researcher bias. Consistent with the COM-B model, responses were grouped into the categories of capability, opportunity, and motivation and links to the TDF domains within the text were also included.

## Results

### Participant characteristics

Altogether 11 pregnant or postpartum women who were overweight or obese participated in the study. Eight (*n* = 8) women were interviewed between gestational weeks 18–33 and three (*n* = 3) women were interviewed during immediate postpartum period 11–20 weeks after the delivery (Table 1). In addition, five (*n* = 5) public health nurses working in maternity clinics (Table 2) participated in the study.

**Table 1:** Characteristics of women who were overweight (*n* = 11)

Characteristics	Women who were overweight (***n*** = 11)
Age (years), median (range)	29 (22–41)
BMI before pregnancy (kg/m^2^), n (%)	
25.0–29.9	3 (27%)
30.0–34.9	7 (64%)
35.0–39.9	0 (0%)
40.0 or more	1 (9%)
Gestational diabetes, n (%)	3 (27%)
Highest educational qualification, n (%)	
Secondary education	6 (55%)
College or University of applied sciences	4 (36%)
University	1 (9%)
Marital status, n (%)	
Married or living with partner	11 (100%)
Planned pregnancy, n (%)	
Yes	9 (82%)
No	1 (9%)
Missing value	1 (9%)

**Table 2:** Characteristics of the public health nurses (*n* = 5)

Characteristics	Public health nurses (***n*** = 5)
Age (years), Median (range)	41 (28–45)
Highest educational qualification, n (%)	
College or University of applied sciences	5 (100%)
Profession, n (%)	
Public health nurse	4 (80%)
Public health nurse and midwife	1 (20%)
Work position, n (%)	
Maternity clinic	2 (40%)
Maternity and child health clinic	3 (60%)
Working experience (years), Median (min-max)	10 (2–22)

### Weight management behaviors of women who were overweight during pregnancy and the postpartum period

The frequency with which the coded phrases corresponded to the COM-B components and TDF domains is reported in Table 3. Most of the data were coded into the TDF domains *knowledge, social and professional role and identity* and *reinforcement*.

**Table 3:** The number of phrases coded under each COM-B component and TDF domain

COM-B component	TDF domain	Number of coded phrases by women who were overweight	Number of coded phrases by public health nurses (focus group interviews)
***Capability***			
Physical capability	*Skills*	17 (3%)	9 (5%)
Psychological capability	*Knowledge*	86 (16%)	24 (12%)
	*Memory, attention and decision processes*	37 (7%)	9 (5%)
	*Behavioral regulation*	25 (5%)	1 (1%)
***Opportunity***			
Physical opportunity	*Environmental context and resources*	20 (4%)	13 (7%)
Social opportunity	*Social influences*	54 (10%)	18 (9%)
***Motivation***			
Automatic motivation	*Reinforcement*	66 (12%)	16 (8%)
	*Emotions*	44 (8%)	19 (10%)
Reflective motivation	*Social/professional role and identity*	93 (17%)	36 (19%)
	*Beliefs about capability*	13 (2%)	20 (10%)
	*Beliefs about consequences*	35 (6%)	18 (9%)
	*Optimism*	11 (2%)	2 (1%)
	*Intentions*	15 (3%)	6 (3%)
	*Goals*	27 (5%)	3 (2%)
Total N of codes		543	194

### Capability – lack of ability to take a practical approach to lifestyle changes

Most participants reported that *physical capability* was not a problem in weight management. However, some women stated that they did not have the *skills* to carry out nutritional advice in practice. Public health nurses also stated that an increasing number of women lacked the skills to make healthy meals or did not know how to follow instructions regarding healthy lifestyles. In terms of *psychological capability* and further *knowledge*, the women who were overweight wanted professional and well-argued information about the risks that obesity posed to themselves and their unborn babies. Some women did not know how to exercise and were uncertain what would be beneficial or harmful during pregnancy or after delivery. Public health nurses reported collecting background information about the women’s everyday situations and their lifestyles and counseled the women individually based on this information. They noticed that some pregnant women lacked knowledge about a healthy lifestyle, such as how to cook healthy food. In addition, the public health nurses reported that they did not necessarily specifically discuss weight; instead, they discussed healthy lifestyle changes and hoped that this would help to reduce weight. Public health nurses wanted concrete and consistent ways to approach the subject.

*“If you could get advice in a different way … not always like, ‘Add vegetables, reduce salt, and reduce sugar.’ … If you just don’t have what it takes to accomplish these things.” (ID4, pregnant woman).*

*“Surprisingly often, you encounter the fact that people have long intervals between meals, and they really eat very rarely, which of course leads to snacking.” (Focus Group 1, public health nurse).*

Reflecting the *memory, attention and decision processes*, most women’s stories contained an apparent indifference towards their weight. Long-lasting overweight seemed to have become a feature of the women’s identity. Some women even felt like outcasts who were not accepted in society. All the women had previous experiences of losing weight; they had tried to change their eating habits and increase exercise, but every time, they failed to reach their goal. Some women had negative experiences during health care encounters because of their weight. Public health nurses also mentioned situations in which women had negative experiences and considered this a barrier to weight management. Furthermore, public health nurses said that they usually asked women’s opinions about the most suitable weight management strategies. With multipara women, they also asked about previous pregnancies and weight management after delivery and utilized approaches that had been effective previously.

*“When you have had a cough for three weeks and sleeping is not so good, the answer is to lose weight. Whatever the problem is, the answer is to lose weight. It is the answer to everything. So … it feels like this world doesn’t belong to you (cries).” (ID4, pregnant woman).*

*“I am at least used to mapping out the situation with the woman herself. I ask whether she has any ideas about why her weight has risen so much, how it feels, and what she has already tried.” (Focus Group 2, public health nurse).*

Regarding *behavioral regulation*, most women who were overweight had experiences of using smart watches to monitor their behavior. From the women’s perspective, the feedback messages and color indicators concerning their physical activity were useful. Some women had kept food diaries and found them effective for self-monitoring eating habits. They thought these different self-monitoring strategies could be utilized in maternity clinics. Public health nurses also considered individualized self-monitoring techniques as concrete tools for broaching the subject. Data from a smartwatch or other device could be utilized in antenatal visits for setting goals and evaluating sleeping and physical activity habits.

*“You could write it down, for example, on your phone, or you could have an app or something where you write down the foods you have eaten during the day. You could keep such a diary every week or a couple of weeks at a time.” (ID7, pregnant woman).*

*“I think, at least, that I have noticed that [smart watches] are quite addictive … when you can follow how you really sleep and move.” (Focus Group 2, public health nurse).*

### Opportunity – difficulties of an immediate commitment to long-term change

Women who were overweight reported pregnancy-related physical discomforts, varying life situations, as well as physical or mental illnesses, as barriers to successful weight management. These challenges reflected their *physical opportunity and environmental context and resources*. Public health nurses emphasized limited time and resources as barriers to weight management counseling; they also felt it was unrealistic to expect immediate lifestyle changes related to eating and exercise during pregnancy. Moreover, time was limited during each antenatal visit, as well as after delivery, and no additional resources existed to guide and support women.

*“I have had to eat quite often all the time, during my whole pregnancy; if I don’t eat, I soon start feeling dizzy.” (ID2, pregnant woman).*

*“After the birth, or …*? *No, not kind of counseling. We just focused on the baby, and the baby’s weight gain … (laughs) At the postnatal check for the mother, she is weighed, and we see if she ​​has reached her pre-pregnancy weight. If she is pretty close or has even reached her pre-pregnancy weight, she will be truly praised.” (Focus Group 1, public health nurse).*

Regarding *social opportunity* and *social influences*, women who were overweight stated that one of the most important aspects of weight management was support from their partners and family. Without support, changing their lifestyle and, by implication, losing weight was considered impossible. One woman described her mother-in-law as constantly baking pastries, making it very hard for her to eat healthily. Peer support was also important. For example, friends in similar life situations or different exercise groups played a major role in weight management support. Public health nurses emphasized the significant role of partners’ as supporters of weight management efforts. They thought it was important to give advice to partners as well, whenever possible. In addition, public health nurses described a lack of peer-support groups for women struggling with weight management.

*“When there is someone who supports me, it is so important to me. When you start to feel like giving up, then there is someone who says, ‘No, do not give up.’” (ID10, postpartum woman).*

*“If the partner visits the maternity clinic with the woman, the partner is encouraged to be involved. You can see that [weight management] is also important for the partner.” (Focus Group 1, public health nurse).*

### Motivation – feeling helpless in the face of overweight

Regarding *automatic motivation* and *reinforcement*, women who were overweight wanted individual and motivating weight management counseling, and they expected to receive feedback about their life habits. Women stated that encouraging them to make small changes and reinforcing their achievements was important. Public health nurses highlighted that the basis of weight management support was listening, the tone of voice, and positive feedback. They also utilized different counseling tools such as pictures of the food pyramid.

*“Of course, the facts must be known … If you have eaten too much, it has to be said, but some kind of future orientation in weight management counseling is needed. It is better to emphasize the positive things, but of course, you can’t go too far, either. You can’t say, ‘Good work! Two bars of chocolate a day!’ (laughs) But constructive and positive but also stimulating counseling is needed so you can actually identify the things you do.” (ID8, pregnant woman).*

*“When the woman describes her daily life and how she is doing, I give positive feedback about her everyday activity. I try to point out the good things and encourage her to continue.” (Focus Group 2, public health nurse).*

Women who were overweight experienced feelings of despair and shame about their weight regarding their *emotions*. Some of them described indifference towards their weight. Denial and defense were the easiest reactions for many of them. Weight management was a very sensitive subject. Public health nurses thought many women who were overweight had low self-esteem and often had psychological reasons to eat, which required counseling in a careful, sensitive and non-stigmatizing manner.

*“They should find a positive way to talk about weight management, so you don’t always feel like crap.”ID4, pregnant woman).*

*“I don’t know whether [weight-management] is hard to broach, but how do you talk about it so she won’t be offended?” Focus Group 1, public health nurse).*

Regarding *reflective motivation*, and more specifically *social/professional role and identity*, women who were overweight expected a more robust stance and professional weight management counseling during pregnancy and after delivery, whereas public health nurses described their roles as advisors of pregnant women. Public health nurses said that they followed protocols for each prenatal visit. They also discussed the health-related risks of being overweight and its consequences for the child. Based on the nurses’ experiences, some women did not want to take responsibility for their own actions regarding weight management. Some pregnant women found it difficult to eat healthy or exercise because they thought that they would gain weight anyway; however, they knew the consequences of their actions. Moreover, some women described the unborn child as their main source of motivation and wanted their children to have a healthy lifestyle. Women also believed that breastfeeding was a way to lose weight, but some of them thought that when a woman breastfeeds, she can eat anything she wants. The unborn baby was not a source of motivation for all women, and in these cases, public health nurses thought it was their professional duty to defend and protect the unborn baby, and they sometimes had to be provocative and arouse emotions. One public health nurse described herself as a baby’s voice shouting, “Don’t do this to me!” From the perspective of the public health nurses, multipara women had better motivation for weight management compared with primiparous women due to their previous experiences of gaining weight during pregnancy and losing weight after delivery, which reflected *beliefs about consequences*.

*“I have gained a lot of weight in a few years, so I have risked my own health, but I don’t want to create those risks for my baby.” (ID5, pregnant woman).*

*“Women are worried about gaining as much weight as they lost after the previous pregnancy. They have put lots of effort into losing weight, and then all of a sudden, they are pregnant again and don’t want all that work to be wasted.” (Focus Group 1, public health nurse).*

Women who were overweight had experiences in which their *intentions* to lose weight usually ended quickly. Some women had intentions to change their lifestyle after the baby was born because they were *optimistic* that they could exercise at the same level as before pregnancy. In addition, women thought that clear *goals* made in collaboration with public health nurses could form a basis for motivation, individual guidance, and support. Furthermore, women who had experience with smart watches suggested that they could make goals for a healthy lifestyle and monitor achieving those goals via the smart watch (i.e., daily physical activity such as steps and activity minutes, diet or a food diary, or weight gain). Women thought that the knowledge that they were being monitored would be motivating in itself. However, contrary to the women’s wishes, public health nurses felt they could not make goals on the women’s behalf. Instead, public health nurses said that they could offer weight management alternatives for the women to choose from. They also had experiences in which women were willing to change but lacked the motivation to make concrete changes.

*“Goal setting supports coping in daily life and motivates you in the bad moments. Goals support me to make the choice to go for a jog rather than stay on the couch.” (ID4, pregnant woman).*

*“The women should set the goals themselves. If the goals are dictated and their actions are controlled by us, it won’t work for longer than a week, if at all.” (Focus Group 1, public health nurse).*

Reflecting on *beliefs about capability*, women felt that changing their lifestyle was quite difficult. They wanted to serve as role models for their children, but they had not lost weight despite their efforts. Public health nurses considered careful mapping of the women’s situation in life extremely important for identifying all factors and possible problems associated with weight management resources. Public health nurses also said that some women did not really want to change their lifestyles.

*“I felt hopeless. I mean, we have a really healthy lifestyle. We eat really healthy, in my opinion: a lot of vegetables. I have always tried to be physically active. But … always, when I go to the scales, no results!” (ID1, postpartum woman).*

*“If there is too much stress in their life, they won’t have the energy to focus on [a healthy lifestyle].” (Focus Group 2, public health nurse).*

The findings regarding identified target behaviors are presented in Table 4.

**Table 4:** Target behaviors categorized by COM-B components and TDF domains

COM-B component	TDF domain	Target behavior
***Capability***		
Physical capability	Skills	Barriers: - Lack of skills needed to cook healthy food - Lack of skills needed to follow instructions for healthy lifestyle choices
Psychological capability	Knowledge	Barriers: - Lack of professional information about the risks of obesity - Lack of concrete counseling on how to eat or exercise - Lack of consistent ways to broach the weight management topic
	Memory, attention and decision processes	Barrier: - Overweight as a feature of women’s identity Facilitator: - Individual weight management counseling Facilitator or barrier: - Previous experiences with weight management
	Behavioral regulation	Facilitator: - Utilizing information from health technology (smart wearables) in antenatal visits
***Opportunity***		
Physical opportunity	Environmental context and resources	Barriers: - Pregnancy related physical discomfort - Lack of time during antenatal visits - Lack of postnatal counseling
Social opportunity	Social influences	Barrier: - Lack of perinatal peer-support groups Facilitators: - Support from partners and family - Peer support
***Motivation***		
Automatic motivation	Reinforcement	Facilitators: - Motivating counseling - Positive feedback - Encouragement to make small changes - Asking questions and listening Facilitator or barrier: - Tone of voice
	Emotions	Barriers: - Despair and feelings of shame - Denial and defense reactions - Low self-esteem - Psychological reasons for eating Facilitator: - Discreet counseling
Reflective motivation	Social/professional role and identity	Barriers: - Conflict: Public health nurses’ self-described role as advisors, which conflicted with women’s expectation of a robust stance and professional counseling - Overweight women’s reluctance to take responsibility for their actions
	Beliefs about capability	Barriers: - Difficult to change lifestyle - Women’s inability to lose weight in the past despite their efforts - Other problems in women’s lives that affect their capabilities - Lack of desire to change their lifestyle on the part of some women Facilitator: - Mapping the women’s life situations
	Beliefs about consequences	Barriers: - Pregnancy as an excuse - Belief that women can eat anything they want while breastfeeding - Public health nurses’ duty to defend and protect the unborn baby Facilitators: - Unborn child as a source of motivation - Positive effects of breastfeeding
	Optimism	Facilitator: - Women’s belief that they could exercise after birth just as they did before pregnancy
	Intentions	Barrier: - Willingness to change but a lack of motivation to make concrete changes Facilitator: - Intentions to change lifestyle after the baby’s birth
	Goals	Barrier: - Paternalistic goals made by public health nurses Facilitator: - Goals made in collaboration with public health nurses

## Discussion

The aim of this study was to identify and describe weight management behaviors from the perspectives of both maternity care professionals and women who were overweight. A theory-based approach was used to identify target behaviors regarding weight management during pregnancy and the postpartum period. Our findings indicate that future weight management interventions for pregnant women should be targeted at providing a consistent way to approach this subject during antenatal visits, increasing women’s knowledge about healthy lifestyles and the supporting the motivation to pursue changes. Furthermore, there is an urgent need to continue to support weight management after childbirth. Public health nurses in maternity clinics need concrete tools to support and motivate women. Health technology and smart wearable devices could constitute one such possibility.

As regards capabilities, it was demonstrated that there is need to find consistent ways to broach the topic of overweight. Both health care professionals and pregnant women seem to avoid discussion regarding obesity and weight-management [27]. Health technology, such as smart wearables could be utilized as part of antenatal care and a means of introducing the topic of weight and weight management. Smart wearables could also support an evaluation of the lifestyle of women who are overweight [15–17].. In line with our findings, the women reported a long history of struggling with weight problems, resulting in overweight becoming a feature of their identity. The women were accustomed to being overweight [11] and had little confidence in themselves anymore. They needed motivating tools and a great deal of support to aid endeavors as regards weight management. Some of the women spoke about negative experiences during previous health care encounters due to their being overweight. Previous studies have also reported similar findings; discussing weight during antenatal visits can be stigmatizing and can make women less receptive to advice or support [12, 28]. These experiences might inhibit women from accepting any attempts to intervene in their weight situation or provide weight management support. Personalized counseling and careful mapping of previous experiences should be promoted. The women who were overweight related that they did not receive enough information about obesity risks, although the public health nurses indicated the opposite. This conflict could be due to overly general counseling or having too many issues to discuss during antenatal visits. Similar to our findings, previous qualitative interview studies have reported inconsistent advice on weight management and knowledge gaps in diet or exercise recommendations during pregnancy [12, 28]. A lack of the skills needed to cook healthy meals and follow a healthy lifestyle was also identified. This could be addressed through technology enabled interventions which could help to develop cooking skills and populate a healthy recipe database [29].

With reference to weight management opportunities, the public health nurses described a lack of resources as the reason for an absence of counseling and support after birth and explained that weight-management was not part of standard care [7, 30]. For women who are overweight resources should be increased postnatally in order to facilitate personalized counseling [11]. We found that one of the important aspects of weight management was support from partners and family. Women’s partners play a significant role in providing advice and are seen as invaluable support [7]. In addition, the women described how their family, relatives, friends, and health care professionals influenced, for example, healthy food choices [31]. This is an important aspect that should be emphasized in future intervention studies and in practice. Furthermore, peer support was considered important. In a previous qualitative study, it was seen as essential to engage women and their partners to discuss weight issues and deliver health counseling sensitively by focusing on individual needs and concerns [32].

In addition to the support from the family, motivation was considered the most important aspect of weight management. In line with previous findings, pregnancy was considered to be an opportunity and motivating period in which to undertake positive lifestyle changes [11, 12]. Although the women were aware of the potential consequences related to poor nutrition or inactivity in pregnancy and expressed interest in wanting to adopt a healthier lifestyle for their child, they found it challenging to engage in a change in behavior during pregnancy. This might be because they believed they would gain weight anyway during pregnancy, so there was no need for a healthy lifestyle. This apparent inconsistency was also found in previous research that reported pregnancy being used as an excuse to indulge [31]. However, some women explained that responsibility for the unborn child was their main reason for being more conscious of their health behavior [31, 33]. The women who were overweight thought that a more uncompromising stance on weight management should be taken by maternity care professionals. Our findings also highlighted that many women who were overweight had low self-esteem, which causes challenges and requires discreet counseling. Low self-esteem might cause avoidance in any attempt to approach the topic of overweight. Respectful and honest communication about body weight can help women feel they have more control over managing their weight [7, 34].

The strength of our theory-based approach was that describing target behaviors enables further tailoring of a structured, evidence-based weight management intervention and related implementation strategies. Using a theoretical framework to understand behavior change will permit future studies to have a more comprehensive assessment of potential barriers and facilitators, as well as the mechanisms linking them to the target behavior. Another strength of our study was that we investigated both women and public health nurses’ experiences and perspectives on weight management during pregnancy overweight. In many domains, the women and public health nurses amplified each other’s observations. However, some conflicts emerged, such as discrepancy between providing and receiving information about obesity risks. It was essential to identify these for utilization in future implementation research. Although the BCW has been widely used in implementation studies in health care settings, most of the previous studies have applied the theory to change in behaviors at organizational or system levels [22]. We utilized the BCW at an individual level to identify practical issues related to weight management behavior. However, our study has some limitations. First, the sample of women who were overweight might have been biased because some of the women might not have felt comfortable discussing this sensitive subject and therefore declined to participate in the study. Second, the data saturation was not completely reached, because the data did not cover all the nuances of the target behaviors. Finally, the participants’ perspectives and experiences might not be transferable to maternity care in other settings, however, previous studies have reported similar findings.

## Conclusions

Our findings offer a theoretical basis to define the contents of the intervention as well as the implementation strategies for future research. The factors identified as barriers to the target behavior, such as a lack of methods to approach the topic of overweight and to motivate women; consistent counseling, especially after birth; and an awareness of the risks associated with unhealthy gestational weight development, could be the topics targeted in future interventions. Facilitators, such as individual and discreet counseling, goal setting, and utilization of support from family and partners, may be employed as intervention components. Moreover, smart wearables and health technology could be a valuable part of an intervention or used as a part of an implementation strategy. Smart wearables and health technology might provide methods to approach the topic of being overweight and a means to support healthy lifestyle choices in maternity care settings.

## Data Availability

The data generated during the current study will not be publicly available due to obligation to maintain confidentiality but are available from the corresponding author on reasonable request.

## Abbreviations

BCW: Behaviour Change Wheel
BMI: Body mass index
BCTTv1: Behaviour Change Technique Taxonomy (v1)
COM-B model: Capability, Opportunity, and Motivation behavior model
TDF: Theoretical Domain Framework
